# General Anaesthesia for Laparoscopic Cholecystectomy in a Patient with the Kearns-Sayre Syndrome

**DOI:** 10.1155/2011/806086

**Published:** 2012-01-15

**Authors:** Paolo Calzavacca, Walter Schmidt, Manuela Guzzi

**Affiliations:** Department of Anaesthesia and Intensive Care, AO Melegnano, PO Uboldo, 20063 Cernsuco sul Naviglio, Italy

## Abstract

We report a case of a 40-year-old man affected by the Kearns-Sayre syndrome who underwent an elective laparoscopic cholecystectomy under general anaesthesia. We describe the management of general anaesthesia in this rare myopathy, with emphasis on the use of rocuronium as muscle blocking agent. Induction was achieved with propofol and fentanyl, and general anaesthesia was maintained with fentanyl and sevoflurane/N_2_O/O_2_ mixture. The anaesthetic plan proved to be safe and effective, and extubation was achieved in the operating theatre. The postoperative recovery of the patient was satisfactory and uneventful.

## 1. Introduction

Kearns-Sayre syndrome (KSS) is a rare myopathy caused by mitochondrial DNA deletions with multisystemic involvement [1]. The syndrome has an estimated incidence of 1 to 3 cases in 100.000 inhabitants [2]. The triad of chronic progressive external ophthalmoplegia, bilateral pigmentary retinopathy, and cardiac conduction abnormalities was first described in a case report of two patients in 1958 by Kearns and Sayre [3]. From a clinical point of view, it is characterised by onset before 20 years of age, ptosis, and the above-mentioned triad constituted of progressive external ophthalmoplegia, pigmentary retinopathy, and cardiac conduction defects. In some cases, cerebellar dysfunction, ataxia, cerebrospinal fluid protein levels > 100 mg/dL, dysphagia, deafness, proximal muscle weakness, intestinal disorders, renal failure, and endocrine abnormalities (hypoparathyroidism, diabetes) can also be present [1–4]. The disease progresses slowly, with new symptoms appearing and previous symptoms worsening.

## 2. Case Report

A 40-year-old, 68 Kg man previously diagnosed to be affected by KSS presented for laparoscopic cholecystectomy at our institution. At a preoperative assessment, the patient was noted to have a trifascicular (first degree atrioventricular, right and left anterior bundle) block on ECG. Holter ECG monitoring revealed occasional supraventricular ectopy. On transthoracic echocardiography, no cardiomyopathy was noted. The ejection fraction was estimated at 55%, and mitral valve prolapse causing mild mitral regurgitation was found. No laboratory test abnormalities were disclosed. No neurologic impairment was noted at the clinical specialist's assessment. Preoperative respiratory function was within normal limits.

The patient had previously undergone general anaesthesia without complications, but the record of that procedure was not available. Preoperative examination revealed a Mallampati score of 2, no predictive factors of difficult airways or impaired ventilation were observed.

The anaesthetic plan included transthoracic pacing (placed before induction), set at 50/min with a capturing threshold at 30 mA. After insertion of a 20 G forearm intravenous cannula and placement of standard monitoring, induction was achieved with fentanyl 100 *μ*g, propofol 140 mg, and rocuronium 25 mg (0.35 mg/Kg). Tracheal intubation was successfully performed after 4 min at the first attempt. A balanced general anaesthesia technique was chosen. Additional fentanyl 100 *μ*g was administered during anaesthesia and hypnosis/anaesthesia was maintained with sevoflurane (maximum 1.6%) and N_2_O/O_2_ mixture 60/40. An episode of sinus bradycardia (heart rate 49/min) was successfully managed with atropine 1 mg. Fluid administration included normal saline 600 mL and acetated ringer's solution 100 mL. Anaesthesia lasted 55 min and the patient was uneventfully extubated in theatre 60 min after induction with no need for reversal as clinical assessment of the patient showed complete recovery from muscle relaxation. Analgesia was provided with paracetamol 1 g i.v. single bolus at the end of surgical procedure, plus continuous infusion of tramadol 150 mg over 24 hours. The patient was then admitted to the intensive care unit (ICU) for planned postoperative monitoring. He was discharged from the ICU on postoperative day 1 pain-free and following an uneventful stay. No abnormalities in the routine postoperative blood tests were observed. Discharge from the hospital was on postoperative day 5.

## 3. Discussion

We report here a case of a patient affected by KSS who was successfully and uneventfully managed with a balanced general anaesthesia plan using rocuronium as muscle relaxant. To our knowledge, this is the first reported case of a KSS patient treated with rocuronium. Mitochondrial diseases are among the most common genetic diseases. Despite this, little is known about these diseases and anaesthesia. In particular, for KSS patients, only a few anecdotal reports are available [4–6]. However, some general and some specific considerations can be made.

Despite a broad spectrum of presentation, all mitochondrial diseases have skeletal muscle weakness which can be exacerbated by general anaesthesia leading to postoperative respiratory failure [6]. Low dose of volatile agents are thus warranted [1]. With the use of volatile agents, lactic acidosis can also result [1]. Such acidosis can be further worsened by the infusion of lactated ringer's solution, which is best avoided. Thus, it seemed appropriate to use a low dose of muscle relaxant to provide good surgical relaxation and to keep at the lowest possible value inhaled anaesthesia.

Specific considerations for KSS patients include [4–7] the risk of complete heart block. Thus, in the pre-operative evaluation, a Holter ECG was performed to rule out major arrhythmic episodes and the planned management included perioperative transthoracic pacing. Moreover, congestive heart failure due to dilated cardiomyopathy [8] can arise because of the cardiodepressant effect of anaesthesia and fluid overload. However, a pre-operative evaluation of cardiac function via echocardiography ruled out cardiomyopathy and attention was given to avoid fluid overload.

Respiratory function also deserves special attention. KSS patients have an increased risk of postoperative respiratory dysfunction both because of prolonged pharmacological neuromuscular blockade and of reduced respiratory drive, which can be further depressed by opioid analgesia [6]. However, adequate analgesia is mandatory in the postoperative period. Accordingly, despite a normal preoperative respiratory function, we planned a postoperative ICU period of observation and monitoring of cardiorespiratory and neurologic function.

Although recommended, neuromuscular monitoring is not always available on daily practice. This is the case at our institution. It is a limitation of our report; however, this can have practiced useful consideration for everyday life. Moreover, sugammadex was not available yet at the time of this report in Italy.

Finally, the associated risk of malignant hyperthermia (MH) remains unproven [9]. However, it seems reasonable to avoid agents that can precipitate MH, in particular, suxamethonium [10]. Volatile agents are also known to be precipitant of MH. Propofol, on the other hand, has never been described as a precipitating agent of MH.

Unusual sensitivity to some induction agents, in particular, to thiopentone and etomidate, has been described [7]. On the other hand, induction with fentanyl and propofol has been reported to be a safe technique [1, 5, 11] in mitochondrial myopathies. Although total intravenous anaesthesia was considered for the procedure, an inhalation anaesthesia with sevoflurane and N_2_O has also been shown to be safe for the maintenance of general anaesthesia [5, 7, 11–13] in those diseases.

Accordingly, we decided to use induction with fentanyl and propofol and maintenance through balanced anaesthesia with sevoflurane and N_2_O. Our case confirms previous reports of efficacy and safety of such a strategy.

The choice of a muscle relaxant took into consideration previous reports and the need for a short-intermediate acting agent. As far as we know, no reports to date have been published using rocuronium as the muscle relaxant in KSS. We decided to use such a muscle-blocking agent at the lowest dosage (0.35 mg/Kg) to facilitate intubation and provide muscle relaxation for surgery in the view of its short-acting action at such a dosage. Moreover, the expected duration of the drug could cover for the duration of the planned surgery with the possibility to provide small top-ups if needed. Atracurium and cisatracurium have similar pharmacodynamic and pharmacokinetic properties and can be valid alternatives. The technique proved to be safe and effective in our case.

In conclusion, we report the first case of a KSS patient successfully and safely treated with rocuronium as paralysing agent for general anaesthesia. We could also confirm previous reports regarding the safety of propofol and fentanyl as induction agents and of sevoflurane and N_2_O as maintenance agents in a case of KSS patient who underwent general anaesthesia.
